# Effects of smoking on the genetic risk of obesity: the population architecture using genomics and epidemiology study

**DOI:** 10.1186/1471-2350-14-6

**Published:** 2013-01-11

**Authors:** Megan D Fesinmeyer, Kari E North, Unhee Lim, Petra Bůžková, Dana C Crawford, Jeffrey Haessler, Myron D Gross, Jay H Fowke, Robert Goodloe, Shelley-Ann Love, Misa Graff, Christopher S Carlson, Lewis H Kuller, Tara C Matise, Ching-Ping Hong, Brian E Henderson, Melissa Allen, Rebecca R Rohde, Ping Mayo, Nathalie Schnetz-Boutaud, Kristine R Monroe, Marylyn D Ritchie, Ross L Prentice, Lawrence N Kolonel, JoAnn E Manson, James Pankow, Lucia A Hindorff, Nora Franceschini, Lynne R Wilkens, Christopher A Haiman, Loic Le Marchand, Ulrike Peters

**Affiliations:** 1 Division of Public Health Sciences, Fred Hutchinson Cancer Research Center, 1100 Fairview Ave N, M4-B402, PO Box 19024, Seattle, WA 98109-1024, USA; 2 Carolina Center for Genome Sciences, School of Public Health, University of North Carolina, Chapel Hill, NC, 27514, USA; 3 Department of Epidemiology, School of Public Health, University of North Carolina at Chapel Hill, Chapel Hill, NC, 27514, USA; 4 Epidemiology Program, University of Hawaii Cancer Center, Honolulu, HI, 96813, USA; 5 Department of Biostatistics, University of Washington, Seattle, USA; 6 Center for Human Genetics Research, Vanderbilt University Medical Center, Nashville, TN, 37203, USA; 7 Department of Laboratory Medicine and Pathology, Medical School, University of Minnesota, Minneapolis, MN, 55455, USA; 8 Vanderbilt Epidemiology Center, Vanderbilt University Medical Center, Nashville, TN, 37203, USA; 9 Department of Medicine, University of Pittsburgh, Pittsburgh, PA, USA; 10 Department of Statistics & Biostatistics, Rutgers University, Piscataway, NJ, 08854, USA; 11 Division of Epidemiology and Community Health, University of Minnesota School of Public Health, Minneapolis, MN, USA; 12 Department of Preventive Medicine, Keck School of Medicine / Norris Comprehensive Cancer Center, University of Southern California, Los Angeles, CA, USA; 13 Brigham and Women’s Hospital, Harvard Medical School, Boston, MA, 02115, USA; 14 Office of Population Genomics, National Human Genome Research Institute, National Institutes of Health, Bethesda, MD, USA

**Keywords:** Obesity, Body mass index, Genome-wide association study, Genetic risk factor, Smoking interactions, Genetic epidemiology

## Abstract

**Background:**

Although smoking behavior is known to affect body mass index (BMI), the potential for smoking to influence genetic associations with BMI is largely unexplored.

**Methods:**

As part of the ‘Population Architecture using Genomics and Epidemiology (PAGE)’ Consortium, we investigated interaction between genetic risk factors associated with BMI and smoking for 10 single nucleotide polymorphisms (SNPs) previously identified in genome-wide association studies. We included 6 studies with a total of 56,466 subjects (16,750 African Americans (AA) and 39,716 European Americans (EA)). We assessed effect modification by testing an interaction term for each SNP and smoking (current vs. former/never) in the linear regression and by stratified analyses.

**Results:**

We did not observe strong evidence for interactions and only observed two interactions with p-values <0.1: for rs6548238/*TMEM18*, the risk allele (C) was associated with BMI only among AA females who were former/never smokers (β = 0.018, p = 0.002), vs. current smokers (β = 0.001, p = 0.95, p_interaction_ = 0.10). For rs9939609/*FTO*, the A allele was more strongly associated with BMI among current smoker EA females (β = 0.017, p = 3.5x10^-5^), vs. former/never smokers (β = 0.006, p = 0.05, p_interaction_ = 0.08).

**Conclusions:**

These analyses provide limited evidence that smoking status may modify genetic effects of previously identified genetic risk factors for BMI. Larger studies are needed to follow up our results.

**Clinical Trial Registration:**

NCT00000611

## Background

The relationship between BMI and smoking is complex. Genetic variation is partially responsible for determining BMI, and genome-wide association studies (GWAS) have identified multiple variants associated with body mass index (BMI) in novel loci [1]. Lifestyle factors also play a key role in determining BMI: smoking affects body fat distribution [2], and current smokers tend to have lower BMI than non-smokers. The mechanism by which smoking regulates adiposity likely involves both appetite suppression via neural pathways [3] and interactions with energy-regulating hormonal feedback loops [4]. The interrelationship between BMI-setting genetic pathways and smoking behavior remains to be explored.

This study examines the potential for effect modification by smoking in the well-replicated association between GWAS-identified SNPs and BMI, using data derived from 56,466 European American (EA) and African American (AA) men and women as part of the NHGRI-supported ‘Population Architecture using Genomics and Epidemiology (PAGE)’ Consortium [5]. We hypothesize that genetic associations with BMI may differ by smoking status. Our results may lead to better understanding of the combined effects of genetics and smoking on obesity among AA and EA men and women.

## Methods

### Study populations

PAGE involves several studies, described in detail elsewhere [5]. PAGE studies included in this analysis are Atherosclerosis Risk in Communities Study (ARIC), Coronary Artery Risk in Young Adults (CARDIA), Cardiovascular Health Study (CHS), Women’s Health Initiative (WHI), Multiethnic Cohort (MEC), and Epidemiologic Architecture for Genes Linked to Environment (EAGLE) accessing the National Health and Nutrition Examination Surveys (NHANES). All studies collected self-identified racial/ethnic group and baseline smoking status via questionnaire. All studies were approved by Institutional Review Boards at their respective sites, and all participants provided informed consent.

To reduce the likelihood of including individuals with extreme BMI due to a comorbid condition or a rare mutation, these analyses only included subjects with BMI ≥18.5 and <70. In our prior analyses, we found that using tighter BMI restrictions (i.e., BMI <40 and BMI <50) did not substantially alter results [6]. A total of 56,466 participants were selected from the PAGE consortium for analysis.

### Anthropometric measurements

In MEC, self-reported height and weight were used to calculate baseline BMI (calculated as weight (kg) ÷ height (m)^2^). Multiple studies have described systematic biases in self-reported compared to measured height and weight; yet in general these differences are small (<1.0 kg/m^2^) and are unlikely to affect any conclusions [7-9]. At all other sites, BMI was calculated from height and weight measured at study enrollment in a clinic setting, with the exception of 140 (0.8%) of the 16,836 WHI subjects whose first available measurements were collected 1 or 3 years after enrollment.

### SNP selection and genotyping

SNPs were selected from GWAS studies published online as of December 31, 2008, based on prior GWAS findings of positive association with BMI or obesity. We analyzed a total of 10 SNPs, after excluding correlated SNPs. Details of the SNP selection process, DNA extraction and genotyping procedures, as well as the association between each of these SNPs and BMI in PAGE have been reported elsewhere [6]. Nine SNPs were analyzed in EA (Table 1), and four SNPs were analyzed in AA (Table 2). We limited our analyses in each group to those SNPs previously associated with BMI in that racial/ethnic group, either in prior GWAS or in our prior report [10]. We also analyzed rs3751812/*FTO* in AA because this SNP has been previously associated with BMI in populations with African ancestry, although our previous analyses were underpowered to detect an association [10,11]. We did not analyze rs3751812/*FTO* in EA because it is strongly correlated with rs9939609/*FTO* in this population.

**Table 1:** Demographic characteristics of PAGE participants, by site, sex, and smoking status

**Current smokers, females**										
	**European Americans**					**African Americans**				
	**total N**	**Mean age (SD)**	**Age range**	**Mean BMI (SD)**	**Max BMI**	**total N**	**Mean age (SD)**	**Age range**	**Mean BMI (SD)**	**Max BMI**
ARIC	1507	53.6 (5.6)	44-66	25.3 (5.0)	55.2	651	52.7 (5.5)	44-65	28.8 (6.1)	52.4
CARDIA	250	25.3 (3.4)	18-30	23.7 (4.0)	37.8	285	25.5 (3.5)	18-30	26.6 (6.1)	45.85
CHS	299	70.4 (4.1 )	65 - 86	25.4 (4.6 )	44.7	68	70.9 (5.3 )	65 - 86	27.2 (5.4)	50.57
EAGLE	688	43.5 (17.0)	18-85	26.8 (6.2)	53.8	500	41.4 (14.7)	18-90	29.5 (7.1)	61.68
MEC	78	56.1 (7.6)	45-71	25.8 (6.4)	52.6	169	58.4 (8.0)	45-75	28.3 (5.8)	48.1
WHI	1010	64.2 (6.6)	50-79	27.4 (6.3)	68.8	423	59.3 (6.5)	50-77	30.9 (7.0)	63.8
				mean BMIpop (SD)					mean BMIpop (SD)	
**Total**	**3832**			26.0 (5.54)		**2096**			29.0 (6.5)	
**Former/never smokers, females**										
	**European Americans**					**African Americans**				
	total N	Mean age (SD)	Age range	Mean BMI (SD)	Max BMI	total N	Mean age (SD)	Age range	Mean BMI (SD)	Max BMI
ARIC	4537	54.1 (5.7)	44-65	27.1 (5.6)	54.7	1977	53.5 (5.8)	44-66	31.5 (6.5)	65.9
CARDIA	716	25.7 (3.3)	18-30	23.3 (4.0)	37.8	676	24.2 (3.9)	18-30	26.6 (6.2)	45.85
CHS	2214	72.6 (5.5)	65 - 100	26.7 (4.9)	48.3	446	73.3 (5.7)	65 - 93	30.1 (5.8 )	58.79
EAGLE	3330	54.3 (18.6)	19-90	27.2 (6.2)	64.5	1656	51.0 (14.7)	23-90	30.1 (6.8)	51.7
MEC	579	58.9 (8.3)	45-76	26.1 (5.6)	56.8	718	60.5 (8.9)	45-77	29.2 (5.9)	56.2
WHI	11865	67.3 (6.8)	50-79	29.1 (6.6)	69.5	3538	61.7 (7.1)	50-79	33.4 (7.7)	68.1
				mean BMIpop (SD)					mean BMIpop (SD)	
**Total**	**23241**			28.1 (6.1)		**9011**			31.6 (7.0)	
**Current smokers, males**										
	**European Americans**					**African Americans**				
	total N	Mean age (SD)	Age range	Mean BMI (SD)	Max BMI	total N	Mean age (SD)	Age range	Mean BMI (SD)	Max BMI
ARIC	1337	54.1 (5.6)	44-65	26.5 (4.0)	56.2	622	53.7 (5.9)	44-66	26.3 (4.8)	45.6
CARDIA	242	25.4 (3.4)	18-30	24.2 (3.4)	34.5	208	25.1 (3.5)	18-30	24.0 (3.8)	36.7
CHS	184	71.3 (4.9)	65 - 90	25.3 (3.3 )	40.7	60	70.0 (4.7 )	65 - 89	25.9 (4.0 )	37.65
EAGLE	859	47.1 (17.7)	18-90	26.8 (5.0)	59.6	544	44.0 (14.9)	18-90	26.2 (5.1)	50.41
MEC	68	59.5 (7.8)	45-76	26.5 (3.8)	37	253	61.2 (7.5)	45-76	26.1 (4.0)	45.7
				mean BMIpop (SD)					mean BMIpop (SD)	
**Total**	**2690**			26.3 (4.3)		**1687**			25.9 (4.7)	
**Former/never smokers, males**										
	**European Americans**					**African Americans**				
	total N	Mean age (SD)	Age range	Mean BMI (SD)	Max BMI	total N	Mean age (SD)	Age range	Mean BMI (SD)	Max BMI
ARIC	4089	55.0 (5.7)	44-66	27.7 (4.0)	53.9	1008	54.0 (6.0)	44-66	28.4 (4.8)	54.4
CARDIA	671	25.6 (3.3)	18-30	24.3 (3.2)	34.5	487	24.0 (3.7)	18-30	25.0 (4.0)	36.7
CHS	1778	73.6 (5.7)	65 - 95	26.6 (3.7)	46.2	245	73.3 (5.8 )	65 - 93	27.2 (4.0 )	38.16
EAGLE	2812	62.5 (16.9)	18-90	27.66 (4.2)	48.4	1310	53.9 (17.1)	19-87	28.6 (5.9)	46.4
MEC	603	61.9 (8.1)	45-77	26.4 (3.8)	49.6	906	63.8 (7.2)	45-77	27.9 (4.2)	51.2
				mean BMIpop (SD)					mean BMIpop (SD)	
**Total**	**9953**			27.0 (3.9)		**3956**			27.5 (4.5)	

*ARIC* Atherosclerosis Risk in Communities Study, *CARDIA* Coronary Artery Risk in Young Adults, *CHS* Cardiovascular Health Study, *EAGLE* Epidemiologic Architecture for Genes Linked to Environment, *MEC* Multiethnic Cohort, *WHI* Women's Health Initiative, *SD* standard deviation, *BMI*: body mass index; Note: minimum BMI was 18.5 for all sites and ancestry groups.

BMIpop: weighted average of mean BMI reported by each PAGE site.

**Table 2:** Complete meta-analysis results in European Americans, effect size expressed in terms of % difference in mean BMI

					**Current smokers**			**Former/never smokers**			Combined sex and smoking status*			
**Gene**	**SNP**	**CA**	**sex**	**p-value for interaction**	**% difference in mean BMI (95% CI)**	**p-value**	**N**	**% difference in mean BMI (95% CI)**	**p-value**	**N**	**% difference in mean BMI (95% CI)**	**p-value**	**N**	**AF**
MTCH2	rs10838738	G	F	0.40	0.6 (-0.4 - 1.51)	0.23	2404	0.4 (-0.2 - 1.11)	0.20	17737	0.51 (0.22 - 0.8)	1.0E-03	34679	0.35
			M	0.24	0.3 (-0.5 - 1.11)	0.40	1525	0.8 (0.1 - 1.41)	0.03	4945				
GNPDA2	rs10938397	G	F	0.87	0.1 (-1.39 - 1.61)	0.88	2165	0.3 (-0.3 - 1.01)	0.28	15961	0.29 (0.004 - 0.69)	0.04	31346	0.43
			M	0.63	0.1 (-1 - 1.31)	0.82	1398	0.5 (-0.2 - 1.21)	0.13	3753				
KCTD15	rs11084753	G	F	0.42	0.8 (0.004 - 1.61)	0.05	3447	−0.1 (-0.7 - 0.4)	0.60	21303	0.11 (-0.18 - 0.4)	6.1E-01	29411	0.67
			M	0.46	−0.1 (-0.7 - 0.6)	0.78	2321	0.2 (-0.3 - 0.7)	0.42	7901				
MC4R	rs12970134	A	F	0.30	0.5 (-0.6 - 1.61)	0.38	1619	0.6 (-0.3 - 1.41)	0.20	14992	0.8 (0.29 - 1.2)	1.3E-03	21987	0.26
			M	0.64	0.1 (-0.9 - 1.11)	0.85	672	1.11 (-0.3 - 2.53)	0.12	2492				
MC4R	rs17782313	C	F	0.60	0.8 (-0.1 - 1.61)	0.09	3449	0.7 (0.1 - 1.31)	0.02	21334	0.22 (-0.004 - 0.51)	0.08	35398	0.22
			M	0.83	0.1 (-0.6 - 0.8)	0.72	2326	0.6 (0.002 - 1.21)	0.05	7930				
NEGR1	rs2815752	T	F	0.45	0.7 (-0.1 - 1.51)	0.07	3393	0.2 (-0.3 - 0.7)	0.38	20898	0.51 (0.11 - 0.8)	9.2E-03	28261	0.63
			M	0.96	0.4 (-0.3 - 1.11)	0.30	2272	0.1 (-0.4 - 0.6)	0.69	7363				
TMEM18	rs6548238	C	F	0.15	1.11 (0.2 - 2.12)	0.02	3424	1.21 (0.5 - 1.82)	7.0E-04	21261	1.02 (0.62 - 1.42)	8.6E-08	37061	0.83
			M	0.69	0.7 (-0.2 - 1.51)	0.12	2290	0.9 (0.2 - 1.51)	0.01	7805				
SH2B1	rs7498665	G	F	0.47	0.7 (-0.2 - 1.61)	0.11	2400	0.9 (0.3 - 1.51)	6.0E-03	17698	0.22 (-0.11 - 0.62)	2.2E-01	31383	0.38
			M	0.49	0.2 (-0.6 - 1.01)	0.67	1530	0.8 (0.1 - 1.51)	0.02	4948				
FTO	rs9939609	A	F	0.08	1.71 (0.9 - 2.53)	3.5E-05	2719	0.6 (-0.01 - 1.11)	0.05	18221	1.31 (1.02 - 1.71)	4.6E-15	28286	0.40
			M	0.94	1.31 (0.6 - 1.92)	2.8E-04	1457	1.51 (1.01 - 2.12)	6.9E-08	5671				

*CA* Coded allele, *M* male, *F* female, *CI* confidence interval, *AF* coded allele frequency.

### Statistical analysis

All analyses were adjusted for continuous age and stratified by racial/ethnic group. Because it has been described that nicotine has antiestrogenic properties and is metabolized differently in men and women, all analyses were additionally stratified by sex [12]. To evaluate effect modification by current smoking, we estimated the association between each SNP and natural log-transformed BMI (lnBMI) in models using linear regression with robust standard errors (SEs) [13], and including a SNP*smoking (current = 1 vs. former/never = 0) interaction term. SNP genotype was coded assuming an additive genetic model (i.e., 0, 1, or 2 copies of the coded allele). We obtained betas specific to current smokers from this model, and to obtain stratum-specific betas for former/never smokers, we re-ran each analysis with a reverse-coded smoking variable (i.e., current = 0 vs. former/never = 1).

To evaluate the association between each SNP and smoking status, we performed logistic regression using current smoking status as the dependent variable (current = 1 vs. former/never = 0).

Results (effect sizes and SEs) from each PAGE study were combined with meta-analysis using R [14]. Fixed-effects meta-analysis was used to calculate effect sizes (β for lnBMI) and 95% confidence intervals (CIs) for each SNP. Within strata defined by racial/ethnic group, smoking status, and sex, we calculated the population mean BMI (BMI_pop_) as the weighted average of mean BMIs reported by each PAGE site (Table 3). We calculated the mean BMI associated with 1 copy of the risk allele with the following formula: BMI_1RA_ = exp ( ln (BMI_pop_) + β ). We then subtracted the BMI_pop_ from BMI_1RA_ to obtain the difference in mean BMI associated with 1 copy of the risk allele. We evaluated I^2^ as a measure of heterogeneity [15], to identify any excess variation between the PAGE cohorts. To address potential population stratification, we repeated analyses for studies that had Ancestry Informative Markers (AIMS) (WHI, ARIC, and MEC, representing >70% of subjects) including the most significant principal components (PCs) derived from AIMs in each model, and compared the results to unadjusted models.

**Table 3:** Complete meta-analysis results for African-Americans, effect size expressed in terms of % difference in mean BMI

					**Current smokers**			**Former/never smokers**			Combined sex and smoking status*			
**Gene**	**SNP**	**CA**	**sex**	**p-value for interaction**	**% difference in mean BMI (95% CI)**	**p-value**	**N**	**% difference in mean BMI (95% CI)**	**p-value**	**N**	**% difference in mean BMI (95% CI)**	**p-value**	**N**	**AF**
GNPDA2	rs10938397	G	F	0.60	0.5 (-0.9 - 1.92)	0.50	1848	1.51 (0.6 - 2.43)	9.2E-04	7664	0.91 (0.3 - 1.41)	1.5E-03	14383	0.24
			M	0.78	1.31 (-0.1 - 2.63)	0.06	1455	1.51 (0.4 - 2.53)	6.9E-03	2831				
MC4R	rs17782313	C	F	0.53	−0.8 (-2.08 - 0.6)	0.27	1758	0.6 (-0.2 - 1.51)	0.15	7365	0.6 (0.03 - 1.11)	4.0E-02	13698	0.29
			M	0.31	−0.1 (-1.29 - 1.21)	0.93	1339	1.21 (0.2 - 2.22)	0.02	2682				
FTO	rs3751812	T	F	0.34	−0.1 (-2.76 - 2.63)	0.94	774	0.4 (-1.09 - 1.92)	0.61	4505	0.4 (-1.11 - 2.01)	0.60	4549	0.12
			M	0.61	2.53 (0.2 - 4.92)	3.0E-02	329	1.51 (-0.3 - 3.25)	0.11	565				
TMEM18	rs6548238	C	F	0.10	0.1 (-1.78 - 2.02)	0.95	1876	1.82 (0.7 - 2.94)	0.002	7707	1.31 (0.6 - 2.01)	2.4E-04	14492	0.88
			M	0.64	2.43 (0.7 - 4.08)	0.01	1463	0.9 (-0.4 - 2.22)	0.18	2855				

*CA* Coded allele, *M* male, *F* female, *CI* confidence interval, *AF* coded allele frequency.

Finally, as a sensitivity analysis to explore the effect of age, we repeated all analyses excluding subjects enrolled in CARDIA, who tended to be younger than subjects enrolled at the other PAGE sites (Table 3).

## Results

Participant demographics and BMI are detailed in Table 3. Analyses included a total of 39,716 EA, and 16,750 AA, with BMI ranging from 18.5 – 69.5 kg/m^2^. Allele frequencies did not differ substantially by smoking status or sex, and thus combined frequencies are presented in all tables. We found no evidence of population stratification, and the sensitivity analysis revealed that excluding subjects enrolled in CARDIA (i.e., younger subjects) did not substantially alter results (data not shown). Thus, we present results unadjusted for PCs, and for all available PAGE subjects (Table 3).

Out of all analyses performed in EA and AAs, none of the SNP*smoking interaction terms were, statistically significant at p-value ≤ 0.05 (Tables 1 and 2). We observed only two interactions that had p-values <0.1: In AA females, the C vs. A allele of rs6548238/*TMEM18* was associated with a 1.82% difference in mean BMI in former/never smokers, compared to a 0.10% difference in mean BMI in current smokers (p_interaction_ = 0.10). In EA men and women, the difference in BMI associated with the C allele of rs6548238/*TMEM18* was very similar in current smokers and former smokers. Rs6548238/*TMEM18* was not associated with smoking status in any of the sex/race groups (data not shown).

Further, we observed that in female EA, the A allele of rs9939609/*FTO* was almost three-fold more strongly associated with BMI in current smokers (1.71% difference in mean BMI) compared with former/never smokers (0.60% difference in mean BMI, (p_interaction_ = 0.08). In EA males, there was no difference in the effect of rs9939609/*FTO* on BMI by smoking status (p = 0.94). The A allele of rs9939609/*FTO* was not associated with current smoking status in either sex (data not shown). As noted above, rs9939609 was not analyzed in AA due to lack of evidence for an association between this SNP and BMI in African ancestry populations.

## Discussion

In our results, we found little evidence for effect modification by smoking status, although two SNPs (rs6548238*/TMEM18* and rs9939609/*FTO)* showed weak evidence for interaction that should be followed up in a larger study. There was some evidence that the BMI-increasing effect of the rs6548238*/TMEM18* C allele was stronger in AA female former/never smokers. The function of *TMEM18* (transmembrane protein 18) is unknown. *TMEM18* is highly expressed in neural tissue, and has been hypothesized to play a role in energy homeostasis via neural pathways controlling food intake [16]. Although *TMEM18* has not been associated with smoking behavior or nicotine metabolism thus far, smoking may modify the effect of *TMEM18* on BMI via energy homeostasis.

The FTO protein may also be involved in neural pathways of energy homeostasis [17]. In our analyses of EA females, the A allele of rs9939609/*FTO* was more strongly associated with BMI among current smokers compared with former/never smokers, although the analysis was underpowered to detect a statistically significant difference between the two groups. If the effect of this SNP truly differs by smoking status, we still cannot determine if smoking affects the function of the risk allele, or if the risk allele attenuates smoking behavior. The latter hypothesis is supported by a study of 6,877 Polish subjects, in which the A allele of rs9939609/*FTO* was associated with older age at smoking initiation and fewer cigarettes smoked per day [18].

Sex-and race/ethnicity-based differences in interaction with smoking could be attributable to differences in smoking behavior, such as cigarette brand choice, and daily vs. occasional “social” smoking [19]. Although we did not have access to such variables, we found no evidence of heterogeneity that would indicate systematic between-group differences.

We did not adjust any analyses to account for multiple testing because we restricted all analyses to SNPs previously known to be associated with BMI. However, these unadjusted results may be prone to increased type 1 error and our results should be replicated in future, larger studies.

## Conclusion

We provide an investigation of the hypothesis that genetic predisposition to obesity may be modified by tobacco use among EA and AA men and women. We observed no strong evidence for SNP*smoking interaction. Despite the relatively large sample size of over 50,000 participants power was limited and future larger studies should investigate the potential sex-specific effects of smoking on each variant’s association with energy balance.

## Competing interests

The authors declare that they have no competing interests.

## Authors’ contributions

MDF, KEN, DCC, MDG, JHF, SL, MG, CSC, LHK, TCM, CPH, BEH, MA, PM, NSB KRM, MDR, RLP, LNK, JEM, JP, LAH, NF, LRW, CAH, LLM, UP participated in the design of the study. DF, UL, PB JH, RG RRR, JP performed the statistical analysis. MDF and UP drafted the manuscript. All authors read and commented on the manuscript and approved the final manuscript.

## Pre-publication history

The pre-publication history for this paper can be accessed here:

http://www.biomedcentral.com/1471-2350/14/6/prepub
